# Extracellular Vesicles in Allergic Rhinitis and Asthma and Laboratory Possibilities for Their Assessment

**DOI:** 10.3390/ijms22052273

**Published:** 2021-02-25

**Authors:** Urszula Demkow, Anna Stelmaszczyk-Emmel

**Affiliations:** Department of Laboratory Diagnostics and Clinical Immunology of Developmental Age, Medical University of Warsaw, 63A Zwirki i Wigury St, 02-091 Warsaw, Poland; urszula.demkow@wum.edu.pl

**Keywords:** asthma, allergy, extracellular vesicles, laboratory techniques, flow cytometry

## Abstract

Currently, extracellular vesicles (EVs) have been implicated in the etiopathogenesis of many diseases, including lung disorders, with the possibility of diagnostic and therapeutic applications. The analysis of EV in respiratory tract diseases faces many obstacles, including material collection from airways, standardization of isolation techniques, detection methods, the analysis of their content, etc. This review focuses on the role of extracellular vesicles in the pathogenesis of atopic respiratory diseases, especially asthma, with a special focus on their clinical applicability as a diagnostic tool. We also summarize available laboratory techniques that enable the detection of EVs in various biological materials, with particular emphasis on flow cytometry. The opportunities and limitations of detecting EV in bronchoalveolar lavage fluid (BALF) were also described.

## 1. Introduction

The group of extracellular vesicles (EVs) consists of exosomes, microvesicles, and apoptotic bodies. Their size is determined at 50–5000 nm. EVs are thought to be responsible for communication between cells in the organism. In recent years, they have become a very popular subject to study in the context of investigating the pathogenesis of various diseases, diagnostics, and with the possibility of therapeutic applications. Their additional advantage is that they can be detected and analyzed in various biological materials collected from the patient. They are assessed in every possible discipline of medicine, from cardiology, hematology to gynecology, as well as neurology. Their role in various lung diseases is also emphasized and studied more and more often. Chronic obstructive pulmonary disease (COPD), lung cancer, pulmonary hypertension, acute lung injury could be mention here, along with atopic respiratory diseases and asthma, which are mentioned in this review [1,2,3,4,5,6].

## 2. Asthma, Allergic Rhinitis, and EVs

### 2.1. Asthma and Allergic Rhinitis

Bronchial asthma, a chronic inflammatory lung disease affecting 5–10% of the global population, is placed among the most common conditions. Asthma is characterized by airway inflammation, reversible airway obstruction, increased mucus production, and bronchial hyperreactivity. A large group of asthmatic patients suffers from an atopic allergy related to T helper 2-type immune response and overproduction of allergen-specific IgE. Allergic asthma and rhinitis are due to a variety of allergens’ sensitization. Both disorders have genetic background skewing the polarization of immune response towards Th2 activation. Asthma patients fall into two categories (endotypes) based on the intensity of Th2 inflammation (Th2 high and Th2 low). The heterogeneity of atopic diseases may also be expressed as differing participation of eosinophils (eosinophilic vs. non-eosinophilic inflammation) and variable clinical manifestations. In the course of early-phase reaction to an allergen, IgE crosslinks high-affinity receptor (FcεRI) on mast cells and basophils, triggering a signaling cascade of intracellular events leading to the activation, degranulation, and the release of a large panel of proinflammatory mediators like histamine, arachidonic acid metabolites, chemokines, cytokines. This specific IgE-driven response is followed by late-phase due to the recruitment of eosinophils, mucus overproduction, and T cell activation. The Th2 cells, by IL-4 and IL-13 acting in concert, support further production of IgE synthesis and, together with IL-5, induce local and systemic eosinophilia perpetuating inflammatory response. Furthermore, a Th2 response leads to the generation of IgE memory B cells [7,8,9].

### 2.2. EVs in General

EVs are released by every cell type, including structural (airway epithelial cells) and immune cells participating in asthmatic inflammation and mediate intercellular communication perpetuating inflammatory reactions. EVs were found to be produced by dendritic cells, monocytes, lymphocytes, mast cells, eosinophils. Most EVs have been shown to contain miRNA, DNA, lipids, and proteins like cytokines, chemokines, adhesion molecules, and proteases. EVs are believed to transfer molecules between cells and express immune-relevant surface molecules such as HLA-DR or co-stimulatory molecules participating in asthmatic inflammation. EVs possess the ability to bind to the surface of cells in order to modulate their functions [10]. All categories of EVs can be found in different body fluids such as serum, bronchoalveolar lavage fluid (BALF), and nasal lavage fluid [11,12]. Many authors started to highlight that EVs can provide novel insights into the pathomechanisms of asthma, including their applicability as diagnostic markers and eventually as a drug-delivery system [3,5,13]. Levänen et al. confirmed that miRNA cargo of EVs in BALF of asthmatic patients differed from healthy controls, and some miRNAs strongly correlated with impaired lung function [14].

### 2.3. EVs from Epithelial Cells

Bartel et al. reported for the first time that lung epithelial cells are able to release EVs apically and basally. Moreover, epithelial cells in the surrounding milieu supplemented with exogenous IL-13, released EVs depleted in miR-92b, miR-34a, and miR-210. Accordingly, decreased content of miR-34a, miR-92b, and miR-210 levels in EVs in nasal lavages from children was associated with airway obstruction, which is a hallmark of asthma [15]. In accord with this observation is the fact that miRNAs are believed to be involved in regulating Th2 differentiation and DC maturation [16]. Bartel et al. suggest that airway epithelial EVs carrying miRNA may play a role in the development of asthma. These authors also confirmed that EVs carrying miRNAs can be found in nasal lavage fluid, being minimally invasive to collect, which is crucial when the examining of pediatric populations is considered [15]. Other scientists, in regards to an experimental model of ovalbumin-induced asthma in mice, state that IL-13 is able to induce mi-RNA-21, exhibiting a significant effect on airway hyperreactivity [17,18]. Mi-RNA-21 also displayed an effect on the development of Th2 cells from primary lymphocytes. This observation corresponds with the statement that mi-RNA-21 plays a role in a cocktail of inflammatory factors in asthma. Therefore mi-RNA-21 can be considered as a biomarker of asthmatic inflammation. [18]. Further studies are warranted to confirm this observation. 

### 2.4. EVs from Immune Cells

Recently different authors unanimously claim that EVs and their cargo are responsible for priming and activation of various inflammatory cells in asthma [5]. Most of the immune cells, such as dendritic cells, lymphocytes T and B, mast cells, eosinophils, and epithelial cells, were proved to use EVs as tools to communicate within the respiratory tract. The bronchial epithelial cells, by miRNA, can directly activate DC and further enhance allergen-specific Th2 polarization [16]. For example, miR-34a enclosed in EVs are found to be involved in DC function and Th2 polarization [19]. The EVs are released from immune cells by cytokines and oxidative stress activators [20]. It was also determined that miRNA from exosomes regulate DC function and maturation via targeting the Wingless/Integrase (Wnt) pathway [21]. Of note is the finding of Vallhov et al. that EVs released by DCs, carrying major cat allergen Fel d 1 together with co-stimulatory molecules and MHC class II antigens, have the ability to modulate the function of lung Th2 lymphocytes [22]. Huang et al. again highlight the important role of miRNA-34a in DC development and their further cooperation with lymphocytes. A decrease in miR-34a in airway epithelial EVs is therefore believed to indirectly modulate CD4+ cells activation [23]. Although not unexpected, mast cells, as important players in the concert of the inflammatory orchestra in asthmatic lungs, are a source of EVs. Skokos et al. suggest that EVs from mast cells may play a role in the recruitment of B and T cells to the lungs [24]. Furthermore, Xie et al. reported that mast cell exosomes carrying FcεRI could suppress allergic reactions by binding to free IgE. The study demonstrated that exosomes derived from mice bone marrow mast cells could bind to free IgE via FcεRI, possess anti-IgE effects, decrease IgE levels and inhibit mast cell activation [25]. Eldh et al. show that exosomes from mast cells attenuate the oxidative stress within the lung. These authors reported that EVs released by mouse mast cells exposed to oxidative stress had a different assortment of mRNA and, as a consequence, the recipient cells become resistant to further oxidative damage; however, the mechanism of this is phenomenon is far from being elucidated [26]. Kumar et al. discovered the link between let-7 miRNAs, and respiratory inflammation, IL13 secretion, and airway hyperactivity after a methacholine challenge. Let-7 miRNAs in the murine lung significantly decreased IL-13 levels in lung tissue, BALF, and serum together with significant improvement of airway hyperreactivity [17]. EVs in serum from severe asthma patients carry higher content of miR-140-3p compared to healthy controls or patients with mild/moderate symptoms. Therefore Suzuki et al. suggest that this marker could have a prognostic value. These authors, using next-generation sequencing, also found significant differences between miRNA profile in EVs from patients with a severe form of the disease as compared to the patients with mild/moderate course [27]. Eosinophils contribute to the late phase of atopic reactions. These cells, acting via EVs, modulate the functions of structural and immune cells of the lung. Eosinophil-derived EVs are potent source proinflammatory factors, i.e., nitric oxide, reactive oxygen species, and express chemotactic factors such as ICAM-1 and integrin a2 [12]. 

### 2.5. Potential Diagnostic and Therapeutic Use of EVs

Recently described alterations of EVs content in asthma might point to the potential use of the exosomes as diagnostic markers and monitoring tools. In a Letter to the Editor, Bahmer et al. showed that total miRNA expression in plasma EVs in patients with well-controlled asthma is the same as in controls, while miR-122-5p was increased in plasma and sputum supernatant EVs in patients with severe asthma [28]. 

Finally, we may speculate about their use as therapeutics [29]. Numerous anti-asthmatic drugs currently approved for asthma therapy do not have the potential to fully control the disease and have important side effects in the course of long-term therapy. Accordingly, the EVs may be used as a delivery system for various anti-inflammatory components. For example, let-7-miRNAs delivered via EVs have the potential for suggesting novel therapeutic intervention. Moreover, Cruz et al. demonstrated that MSC-derived EVs abrogated allergic respiratory tract inflammatory reactions in immunocompromised mice induced by exposures to *Aspergillus* extract [30]. The same directionality of effect was observed by de Castro et al., who found that MSC-derived EVs decreased inflammatory reaction and inhibited tissue remodeling in ovalbumin-treated mice [31]. 

## 3. Laboratory Techniques

Despite their enormous potential to become useful biomarkers for diagnosing, predicting, and monitoring various disease entities, EVs are not largely used in clinical medicine as they are difficult to assess in a laboratory. Accordingly, specific methods for their identification and characterization, sufficient standardization of the pre-laboratory phase, and protocols for the preparation and storage of clinical material need to be developed [2,32,33,34,35].

To face this problem, the first Minimal information for studies of extracellular vesicles 2014 (MISEV2014), a position statement of the International Society for Extracellular Vesicles, was published in 2014, followed by an update to the MISEV2014 guidelines in 2018. These documents opened a way to the standardization of the nomenclature and the recommendation for the collection and processing of the clinical samples, as well as identification, counting, and characterization of EVs [36,37].

The authors of the guidelines were fully aware of the limitations of the document based on what was reflected on in the conclusion: “Finally, there are exceptions to every rule. MISEV2018 is meant to guide and improve the field, not stifle it. If MISEV recommendations and requirements cannot be met, authors will then need to explain their unique situation and describe their attempts to meet the guidelines and the reason for failure. These guidelines will also continue to evolve” [37].

Further studies are warranted to establish the physiological relevance and the diagnostic potential of EVs in different clinical materials. There is still a long way to elaborate, standardize and validate EV tests for clinical use.

## 4. Collection, Preparation, and Storage of Material for EVs Assessment in Asthma and Allergic Rhinitis

A very important aspect of EVs assessment is the preanalytical phase, i.e., preparation of the collected sample for testing—used anticoagulant, centrifugation, and storage conditions. Different protocols used in the preanalytical phase may result in obtaining different results [32,34].

The material most commonly used to explore respiratory allergy and asthma in BALF. There are also scarce publications pointing to the applicability of nasal lavage [38] or exhaled breath condensate [39] as valid clinical material for EVs detection in patients with asthma or respiratory allergies.

BALF is collected from patients according to standard protocols used in the given units. Most biological samples collected from patients have different degrees of viscosity and chemical composition than culture-conditioned media; therefore, the protocol should be appropriately adapted. Concerning BALF, the isolation of EVs is confounded with the presence of the surfactant in the same fraction as EVs [37].

The protocol most frequently used to isolate EVs from BALF was described by Thery et al. 2006 [40]. It is used with or without modifications [4,41,42,43,44]. Table 1 summarizes several modifications of the EVs extraction protocol from BALF used by different researchers.

After proper isolation, the characterization of EVs present in BALF can be started. EVs analyzed in patients with asthma are those of known cellular origin, such as mast cell, eosinophil, neutrophil, B-cell, T-cell, dendritic cell, myeloid, platelet, epithelial cell, fibroblast, and mesenchymal cell-derived EVs. In BALF, EVs from epithelial cells and from immune cells are most often delt with [3,5,42,44].

The following antigens and factors are usually assessed in BALF EVs: CD63, CD54, CD81, CD9, CD36, HLA-DR, pro- and anti-inflammatory cytokines, leukotrienes as well as miRNAs and lipids [4,15,41,42,44].

## 5. Methods for the Detection of EVs from Specific Tissues or Cells

Various methods are used for the analysis of EVs in laboratories. Most of the applied techniques are based on fluorescent methods. These include NTA (nanoparticle tracking analysis), flow cytometry, fluorescence microscopy, super-resolution microscopy, quantitative PCR, microfluidics, and microarrays. All of these have their advantages and limitations [33].

Here, we focus on a sensitive and reproducible method for EVs flow cytometry detection that allows characterization of small amounts of microparticles in a routine laboratory. Flow cytometry enables the detection of EVs in the tested sample, as well as to determine the size and phenotype of particles in a short time [45,46].

The difficulties in the detecting of EVs are their small size—most often below 200 nm, and the presence of very different composition together with concomitant particles of similar size [45].

The analyzers used for EVs assessment by flow cytometry fall into three categories:

1. Clinical flow cytometers, used primarily to perform tests on white and red blood cells, but with proper preparation of the analyzer and setting the reading parameters to enable the evaluation of EVs.
2. Nanoparticles flow cytometers—specially designed for the detection and identification of small particles, EVs below 100 nm, but not widely used in laboratories
3. Imaging flow cytometers.

### 5.1. Clinical Flow Cytometers

Clinical flow cytometers are dedicated to evaluating particles with sizes around 200–500 nm. The antigens to be detected are localized by monoclonal antibodies conjugated with fluorochromes. The particles move in the liquid stream and individually pass through the beam of laser light. At this point, the excitation of fluorochromes and the emission of light of a certain length takes place. Cytometry also allows for the assessment of the relative size and granularity of cells through forward and side scatter analysis. The signal is converted into a digital form, and the appropriate software gives researchers a chance for a detailed analysis of the obtained results. Depending on the equipment (the number of available lasers and detectors) and the appropriate setting of the reading parameters in most of the currently used cytometers, can evaluate about 10–12 different antigens per molecule [47,48,49].

**Figure 1:**
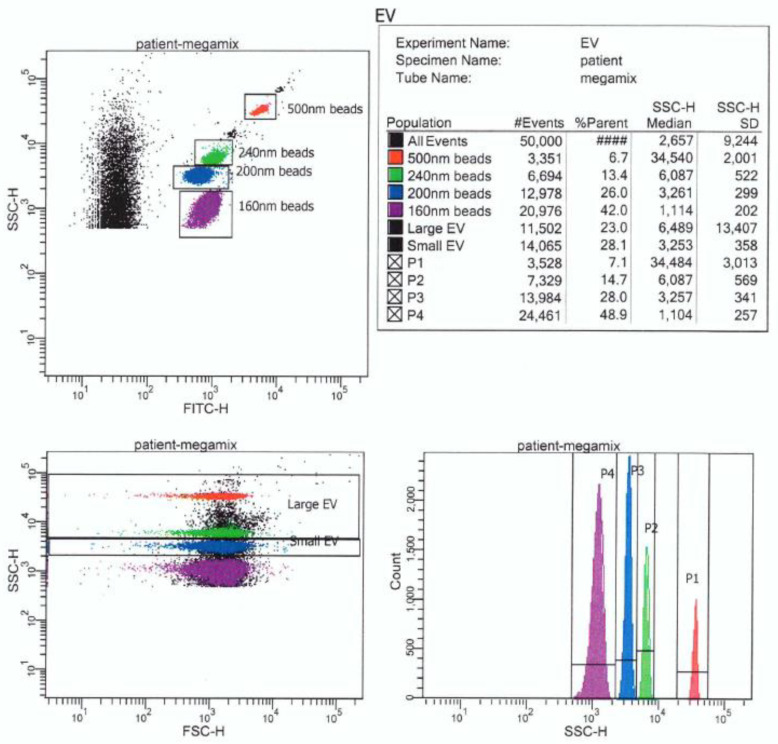
Example of dot plots and histogram of megamix-Plus SSC polystyrene beads using DIVa 8.0.1 software and FACSCantoII flow cytometer (Becton, Dickinson and Company) (own results, unpublished).

In order to facilitate and validate the analysis of small particles, fluorescent beads of different diameters are attached to the samples taken. This enables the appropriate setting of the reading parameters for the EVs analysis. Figure 1 shows an example of polystyrene beads analysis (own results, unpublished).

The fluorescent beads include polystyrene beads (megamix, megmix-Plus FSC (forward scatter), megamix-Plus SSC (side scatter)—presented in Figure 1), sub-micrometer polystyrene beads, silica beads (ApogeeMix), hollow organosilica beads (HOBs, available in two sizes). However, the use of polystyrene beads or silica beads does not allow for efficient calibration and sizing of the EVs because they have different light scattering intensities than EVs. So far, the best feature for this purpose has been demonstrated by HOBs [50]. Only these beads are considered to enable calibration and evaluation of the EVs size [45].

Generally available computer software (FCMpass and Rosetta Calibration) [51,52] have been developed to enable the evaluation of the EVs and thus the better performance of the EVs evaluation analysis on the cytometer. The standardization of EV detection and good performance characteristics of the tests with the use of a cytometer is still to be established. In addition to the previously mentioned MISEV2014 and MISEV2018, other projects also focus on the standardization of EV assessment by flow cytometry (METVESII—metves.eu). In 2019, the EV Flow Cytometry Working Group was established by international scientific societies interested in the development of this field in order to improve the standardization and education of researchers. In 2020, MIFlowCyt-EVs, a framework for standardized reporting of EVs flow cytometry experiments [53,54], was released. Further educational articles and seminars for researchers are being prepared (EV Flow Series) [45]. Calibration and standardization attempts based on the appropriate setting of cytometer parameters (fluorescence-triggered) and appropriate analysis are also undertaken by individual research centers [55,56].

**Figure 2:**
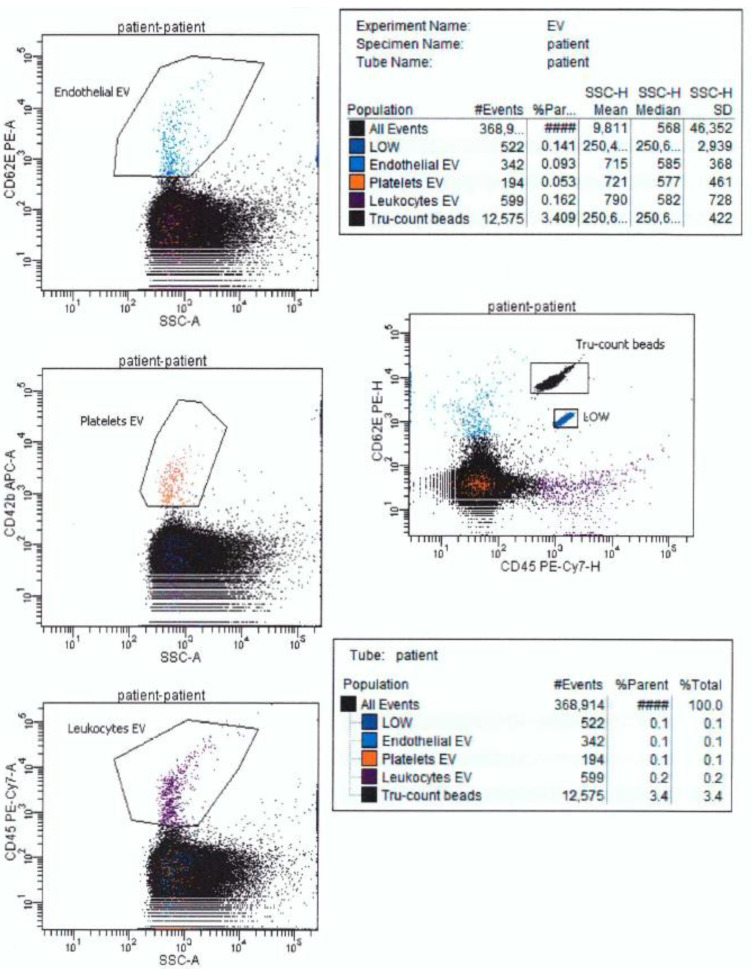

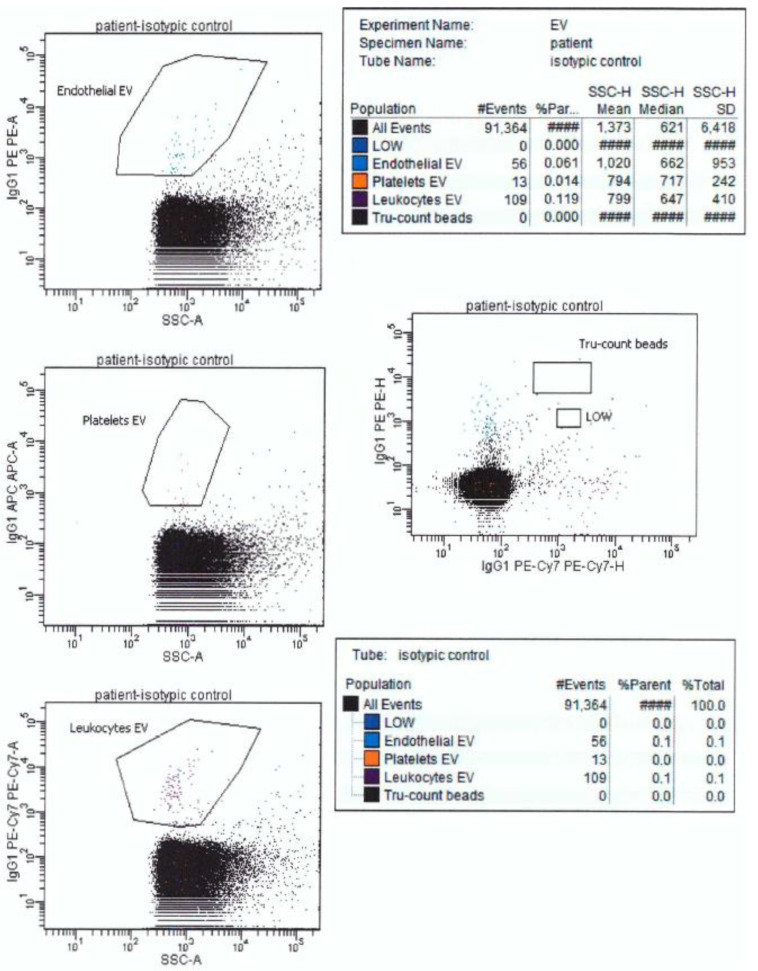
Example of isotypic control and platelets-, endothelial-, leukocytes- EVs with anti-CD42b-APC, anti-CD63-PE, anti-CD45-PE-Cy7 antibodies respectively in Tru-count tube with LOW Tru-count control beads using DIVa 8.0.1 software and FACSCantoII flow cytometer (Becton, Dickinson and Company) (own results, unpublished). As an internal control of the assessments of the absolute number of particles, manufacturer controls were used (LOW control beads were used because the number of detected particles was small).

In cytometry, in addition to establishing the presence of EVs in the biological material, their origin can be determined, depending on the expression of EV antigens. This is possible thanks to the use of monoclonal antibodies [2]. Figure 2 presents the simplest analysis of EVs originating from endothelial cells, platelet and leukocytes in patient plasma using the following antibodies: anti-CD62E PE (for endothelial EVs), anti-CD42b APC (for platelet EVs), and anti-CD45 PE-Cy7 (for leukocyte EVs) [57,58,59].

Only a few flow cytometers are able to assess the absolute number in addition to the qualitative assessment of cells, EVs, and the percentage of molecules with the expression of a given antigen. However, if appropriate counting beads are used to perform the test, it is possible to assess the absolute number of EVs in a given biological material on each cytometer. The results of such an assessment are shown in Figure 2.

Despite the use of selected types of beads, the application of appropriate software for small EVs analysis is still necessary. Apart from the precise setting of the cytometer, the measurement requires extensive experience of the researcher. EVs assessment based on the use of bead-based flow cytometry has been largely introduced for basic and clinical research [60,61]. This technique has been successfully used for the evaluation of various small molecules or proteins, such as cytokines and chemokines. Such a procedure enables the EVs to attach themselves to the beads. The structures obtained at the end are much larger and easier to evaluate by standard cytometers. The above-mentioned technique allows for the detection and qualitative evaluation of EVs surface antigens in various body fluids as well as in cell culture supernatants. At the same time, a large number of different antigens can be detected in one sample, and thus, the mixture of EVs can be defined very well. In the work of Wiklander et al. from 2018, the authors successfully assessed 37 different markers on the EVs surface in one sample. The validated multiplex bead-based flow cytometry methods enable a sensitive, reproducible, and extensive evaluation of EVs. At the same time, they do not require sophisticated equipment or vast researcher experience. They also allow for the use of standard cytometers to detect EVs, which are normally too small to be detected. Recently different researchers highlighted the applicability of the above-mentioned method in oncology [60,61].

### 5.2. Nanoparticles and Imaging Flow Cytometers

The use of EVs provides a valuable insight into pathological processes in the lung. EVs can serve as biomarkers, which require specific methodologies. Nanoparticle flow cytometers have been developed as a potential tool for the detection and analysis of molecules of a smaller size than those assessed in standard cell cytometers [62,63]. Further options to extend the possibilities of flow cytometry include imaging flow cytometers. In addition to all the diagnostic options offered by classical cytometry (fast and high-throughput scatter measurement and many fluorescence markers), it also provides the ability to view images of the molecules being assessed. It is characterized by significantly lower background noise, and it is able to distinguish true single events by imaging objects and excluding convergent events or debris from the analysis. However, just like nanoparticles flow cytometers, imaging flow cytometers are not widely applied and require experience to operate [63,64,65,66].

## 6. Conclusions

As it is evident from the available studies, EVs can be a great source of information about the pathomechanism, diagnostics, monitoring, and therapy of asthma and allergic rhinitis. The first step is to define the normal composition of EVs and to contrast pathological changes with well-defined cut off in BALF. Currently, great differences between various laboratories exist in terms of the standardization of EVs detection and analysis. Accordingly, there is a great need to establish a standardization of protocols and to validate the results across clinical laboratories. Nevertheless, for this to be fully possible, appropriate assays need to be developed to detect and identify EVs. At present, standard flow cytometry can be optimized to detect EVs. However, it still requires a lot of effort to standardize the assay in terms of its sensitivity, specificity, and reproducibility to release exosome-based diagnostics tests for asthma. Finally, exosome research is of considerable interest to both basic scientists and clinicians as it offers new possibilities for substantial progress toward better care for asthma patients. 

## Figures and Tables

**Table 1:** Protocol for extracellular vesicle EV isolation from body fluids by Thery et al. [40] with adaptation to bronchoalveolar lavage fluid (BALF) [41,43,44].

Operation Steps	Thery et al., 2006	Modifications	Purpose of Action
Step 1	Viscous body fluids Dilute fluid with an equal volume of PBS	BALF Centrifugation: 300–500× *g*, 10 min, at 4 °C	To remove airway cells (BALF)
Step 2	Centrifugation: 2000× *g*, 30 min, at 4 °C	Transfer supernatant, remove pellet centrifugation: 2000–3000× *g*, 10 min, at 4 °C	To remove dead cells and large cellular debris
Step 3	Transfer supernatant, remove pellet centrifugation: 12,000× *g*, 45 min, at 4 °C	Transfer supernatant, remove pellet centrifugation: 10,000–16,500× *g*, 30 min, at 4 °C Filter supernatant (0.22 µm)	Again, to remove dead cells and cellular debris
Step 4	Transfer supernatant, centrifugation: 110,000× *g*, 120 min, at 4 °C	Transfer supernatant centrifugation: 100,000–140,000× *g*, 70–130 min, at 4 °C	To have EVs in pellet
Step 5	Discard supernatant, Resuspend pellet in 1 mL PBS Filter the suspension (0.22 µm), centrifugation of the filtrate: 110,000× *g*, 70 min, 4 °C. Discard supernatant, wash pellet in PBS, centrifugation: 110,000× g, 70 min, 4 °C. Pellet resuspended in PBS, stored at −80 °C	Discard supernatant, wash pellet in PBS, centrifugation: 100,000–110,000× *g*, 70 min, at 4 °C. Pellet resuspended in PBS, stored at −80 °C	To remove any contaminating proteins and to have EVs in the pellet

Min-minutes, PBS-phosphate buffered saline, supernatant-fluid above pellet after material centrifugation.

## Data Availability

Not applicable.

## References

[1] Witwer K.W., Théry C. (2019). Extracellular vesicles or exosomes? On primacy, precision, and popularity influencing a choice of nomenclature. J. Extracell. Vesicles.

[2] Wen C., Seeger R.C., Fabbri M., Wang L., Wayne A.S., Jong A.Y. (2017). Biological roles and potential applications of immune cell-derived extracellular vesicles. J. Extracell. Vesicles.

[3] Mohan A., Agarwal S., Clauss M., Britt N.S., Dhillon N. (2020). Extracellular vesicles: Novel communicators in lung diseases. Respir. Res..

[4] Wu F., Yin Z., Yang L., Fan J., Xu J., Jin Y., Yu J., Zhang D., Yang G. (2019). Smoking Induced Extracellular Vesicles Release and Their Distinct Properties in Non-Small Cell Lung Cancer. J. Cancer.

[5] Sangaphunchai P., Todd I., Fairclough L.C. (2020). Extracellular vesicles and asthma: A review of the literature. Clin. Exp. Allergy.

[6] Desrochers L.M., Antonyak M.A., Cerione R.A. (2016). Extracellular Vesicles: Satellites of Information Transfer in Cancer and Stem Cell Biology. Dev. Cell.

[7] Papi A., Brightling C., Pedersen S.E., Reddel H.K. (2018). Asthma. Lancet.

[8] Mandlik D.S., Mandlik S.K. (2020). New perspectives in bronchial asthma: Pathological, immunological alterations, biological targets, and pharmacotherapy. Immunopharmacol. Immunotoxicol..

[9] Calvén J., Ax E., Rådinger M. (2020). The Airway Epithelium-A Central Player in Asthma Pathogenesis. Int. J. Mol. Sci..

[10] Yáñez-Mó M., Siljander P.R., Andreu Z., Zavec A.B., Borras F.E., Buzas E.I., Buzas K., Casal E., Cappello F., Carvalho J. (2015). Biological properties of extracellular vesicles and their physiological functions. J. Extracell. Vesicles.

[11] Simpson R.J., Jensen S.S., Lim J.W. (2008). Proteomic profiling of exosomes: Current perspectives. Proteomics.

[12] Bartel S., Deshane J., Wilkinson T., Gabrielsson S. (2020). Extracellular Vesicles as Mediators of Cellular Cross Talk in the Lung Microenvironment. Front. Med..

[13] Gelfand E.W., Joetham A., Wang M., Takeda K., Schedel M. (2017). Spectrum of T-lymphocyte activities regulating allergic lung inflammation. Immunol. Rev..

[14] Levänen B., Bhakta N.R., Torregrosa Paredes P., Barbeau R., Hiltbrunner S., Pollack J.L., Skold M., Svartengren M., Grunewald J., Gabrielsson S. (2013). Altered microRNA profiles in bronchoalveolar lavage fluid exosomes in asthmatic patients. J. Allergy Clin. Immunol..

[15] Bartel S., La Grutta S., Cilluffo G., Perconti G., Bongiovanni A., Giallongo A., Behrends J., Kruppa J., Hermann S., Chiang D. (2020). Human airway epithelial extracellular vesicle miRNA signature is altered upon asthma development. Allergy.

[16] Stumpfova Z., Hezova R., Meli A.C., Slaby O., Michalek J. (2014). MicroRNA profiling of activated and tolerogenic human dendritic cells. Mediat. Inflamm..

[17] Kumar M., Ahmad T., Sharma A., Mabalirajan U., Kulshreshtha A., Agrawal A., Ghosh B. (2011). Let-7 microRNA-mediated regulation of IL-13 and allergic airway inflammation. J. Allergy Clin. Immunol..

[18] Lu T.X., Munitz A., Rothenberg M.E. (2009). MicroRNA-21 is up-regulated in allergic airway inflammation and regulates IL-12p35 expression. J. Immunol..

[19] Li Y.-Y., Tao Y.-W., Gao S., Li P., Zheng J.-M., Zhang S.-E., Liang J., Zhang Y. (2018). Cancer-associated fibroblasts contribute to oral cancer cells proliferation and metastasis via exosome mediated paracrine miR-34a-5p. EBioMedicine.

[20] Fujita Y., Kosaka N., Araya J., Kuwano K., Ochiya T. (2015). Extracellular vesicles in lung microenvironment and pathogenesis. Trends Mol. Med..

[21] Hashimi S.T., Fulcher J.A., Chang M.H., Gov L., Wang S., Lee B. (2009). MicroRNA profiling identifies miR-34a and miR-21 and their target genes JAG1 and WNT1 in the coordinate regulation of dendritic cell differentiation. Blood.

[22] Vallhov H., Gutzeit C., Hultenby K., valenta R., Groenlund H., Scheynius A. (2015). Dendritic cell-derived exosomes carry the major cat allergen Fel d 1 and induce an allergic immune response. Allergy.

[23] Huang A., Yang Y.I., Chen S.I., Xia F., Sun D., Fang D., Xiong S., Jin L., Zhang J. (2017). MiR-34a promotes DCs d evelopment and inhibits their function on T cell activation by targeting WNT1. Oncotarget.

[24] Skokos D., Le Panse S., Villa I., Pousselle J.S., Peronet R., Namane A., David B., Mecheri S. (2001). Nonspecific B and T cell-stimulatory activity mediated by mast cells is associated with exosomes. Int. Arch. Allergy Immunol..

[25] Xie G., Yang H., Peng X., Lin L., Wang J., Lin K., Cui Z., Li J., Xiao H., Linag Y. (2018). Mast cell exosomes can suppress allergic reactions by binding to IgE. J. Allergy Clin. Immunol..

[26] Eldh M., Ekström K., Valadi H., Sjostrand M., Olsson B., Jernas M., Lotvall J. (2010). Exosomes communicate protective messages during oxidative stress; possible role of exosomal shuttle RNA. PLoS ONE.

[27] Suzuki M., Konno S., Makita H., Shimizu K., Kimura H., Kimura H., Nishimura M. (2016). Altered circulating exosomal RNA profiles detected by next-generation sequencing in patients with s evere asthma. Eur. Respiratory Soc..

[28] Bahmer T., Krauss-Etschmann S., Buschmann D., Behrends J., Watz H., Kirsten A.-M., Pedersen F., Waschki B., Fuchs O., Pfaffl M.W. (2020). RNA-seq-based profiling of extracellular vesicles in plasma reveals a potential role of miR-122-5p in asthma. Allergy.

[29] Worthington E.N., Hagood J.S. (2020). Therapeutic Use of Extracellular Vesicles for Acute and Chronic Lung Disease. Int. J. Mol. Sci..

[30] Cruz F.F., Borg Z.D., Goodwin M., Sokocevic D., Wagner D.E., Coffey A., Antunes M., Robinson K.L., Mitsialis S.A., Kourembanas S. (2015). Systemic Administration of Human Bone Marrow-Derived Mesenchymal Stromal Cell Extracellular Vesicles Ameliorates Aspergillus Hyphal Extract-Induced Allergic Airway Inflammation in Immunocompetent Mice. Stem. Cells Transl. Med..

[31] de Castro L.L., Xisto D.G., Kitoko J.Z., Cruz F.F., Olsen P.C., Redondo P.A.G., Ferreira T.P.T., Weiss D.J., Martins M.A., Morales M.M. (2017). Human adipose tissue mesenchymal stromal cells and their extracellular vesicles act differentially on lung mechanics and inflammation in experimental allergic asthma. Stem. Cell Res. Ther..

[32] Yuana Y., Bertina R.M., Osanto S. (2011). Pre-analytical and analytical issues in the analysis of blood microparticles. Thromb. Haemost..

[33] Panagopoulou M.S., Wark A.W., Birch D.J.S., Gregory C.D. (2020). Phenotypic analysis of extracellular vesicles: A review on the applications of fluorescence. J. Extracell. Vesicles.

[34] Coumans F.A.W., Brisson A.R., Buzas E.I., Dignat-George F., Drees E.D.D., El-Andaloussi S., Emanueli C., Gasecka A., Hendrix A., Hill A.F. (2017). Methodological guidelines to study extracellular vesicles. Circ. Res..

[35] Yuana Y., Böing A.N., Grootemaat A.E., van der Pol E., Hau C.M., Cizmar P., Buhr E., Sturk A., Nieuwland R. (2015). Handling and storage of human body fluids for analysis of extracellular vesicles. Extracell. Vesicles.

[36] Lotvall J., Hill A.F., Hochberg F., Buzas E.L., Di Vizio D., Gardiner C., Gho Y.S., Kurochkin I.V., Mathivanan S., Quesenberry P. (2014). Minimal experimental requirements for definition of extracellular vesicles and their functions: A position statement from the international society for extracellular vesicles. J. Extracell. Vesicles.

[37] Théry C., Witwer K.W., Aikawa E., Alcaraz M.J., Andreson J.D., Andriantsitohaina R., Antoniou A., Arab T., Archer F., Atkin-Smith G.K. (2018). Minimal information for studies of extracellular vesicles 2018 [MISEV2018]: A position statement of the International Society for Extracellular Vesicles and update of the MIS ev2014 guidelines. J. Extracell. Vesicles.

[38] Lässer C., O’Neil S.E., Shelke G.V., Sihlbom C., Hansson S.F., Gho Y.S., Lundback B., Lotvall J. (2016). Exosomes in the nose induce immune cell trafficking and harbour an altered protein cargo in chronic airway inflammation. J. Transl. Med..

[39] Sinha A., Yadav A.K., Chakraborty S., Kabra S.K., Lodha R., Kumar M., Kulshreshtha A., Sethi T., Pandey R., Malik G. (2013). Exosome-enclosed microRNAs in exhaled breath hold potential for biomarker discovery in patients with pulmonary diseases. J. Allergy Clin. Immunol..

[40] Théry C., Amigorena S., Raposo G., Clayton A. (2006). Isolation and characterization of exosomes from cell culture supernatants and biologicalfluids. Curr. Protoc. Cell Biol..

[41] Héliot A., Landkocz Y., Roy Saint-Georges F., Gosset P., Billet S., Shirali P., Courcot D., Martin P.J. (2017). Smoker extracellular vesicles influence status of human bronchial epithelial cells. Int. J. Hyg. Environ. Heal..

[42] Torregrosa Paredes P., Esser J., Admyre C., Nord M., Rahman Q.K., Lukic A., Radmark O., Groenneberg R., Grunewald J., Eklund A. (2012). Bronchoalveolar lavage fluid exosomes contribute to cytokine and leukotriene production in allergic asthma. Allergy.

[43] Qazi K.R., Torregrosa Paredes P., Dahlberg B., Grunewald J., Eklund A., Gabrielsson S. (2010). Proinflammatory exosomes in bronchoalveolar lavage fluid of patients with sarcoidosis. Thorax.

[44] Hough K.P., Wilson L.S., Trevor J.L., Strenkowski J.G., Maina N., Kim Y.-I., Spell M.L., Wang Y., Chanda D., Rodriguez Dager J. (2018). Unique Lipid Signatures of Extracellular Vesicles from the Airways of Asthmatics. Sci. Rep..

[45] Kuiper M., van de Nes A., Nieuwland R., Varga Z., van der Pol E. (2021). Reliable measurements of extracellular vesicles by clinical flow cytometry. Am. J. Reprod. Immunol..

[46] Nolan J.P. (2015). Flow cytometry of extracellular vesicles: Potential, pitfalls, and prospects. Curr. Protoc. Cytom..

[47] Cossarizza A., Chang H.-D., Radbruch A., Acs A., Adam D., Adam-Klages S., Agace W.W., Aghaeepour N., Akdis M., Allez M. (2019). Guidelines for the use of flow cytometry and cell sorting in immunological studies (second edition). Eur. J. Immunol..

[48] Shapiro H.M. (2003). Practical Flow Cytometry.

[49] Adan A., Alizada G., Kiraz Y., Baran Y., Nalbant A. (2017). Flow cytometry: Basic principles and applications. Crit. Rev. Biotechnol..

[50] Varga Z., Van der Pol E., Pálmai M., Garcia-Diez R., Gollwitzer C., Krumrey M., Frajkin J.-L., Gasecka A., Hajji N., van Leeuwen T.G. (2018). Hollow organosilica beads as reference particles for optical detection of extracellular vesicles. J. Thromb. Haemost..

[51] Welsh J.A., Horak P., Wilkinson J.S., Ford V.J., Jones J.C., Smith D., Holloway J.A., Englyst N.A. (2019). FCMPASS software aids extracellular vesicle light scatter standardization. Cytom. Part A.

[52] van der Pol E., Sturk A., van Leeuwen T., Nieuwland R., Coumans F. (2018). ISTH-SSC-VB Working Group Standardization of extracellular vesicle measurements by flow cytometry through vesicle diameter approximation. J. Thromb. Haemost..

[53] Welsh J.A., Van Der Pol E., Arkesteijn G.J.A., Bremer M., Brisson A., Coumans F., Dignat-George F., Doggan E., Ghiran I., Giebel B. (2020). MIFlowCyt- ev: A framework for standardized reporting of extracellular vesicle flow cytometry experiments. J. Extracell. Vesicles.

[54] Welsh J.A., Tang V.A., van der Pol E., Gorgens A. (2020). MIFlowCyt-EV: The Next Chapter in the Reporting and Reliability of Single Extracellular Vesicle Flow Cytometry Experiments. Cytom. Part A.

[55] Simeone P., Celia C., Bologna G., Ercolino E., Pierdomenico L., Cilurzo F., Grande R., Diomede F., Vespa S., Canonico B. (2020). Diameters and Fluorescence Calibration for Extracellular Vesicle Analyses by Flow Cytometry. Int. J. Mol. Sci..

[56] Oesterreicher J., Pultar M., Schneider J., Muehleder S., Zipperle J., Grillari J., Holnthoner W. (2020). Fluorescence-Based Nanoparticle Tracking Analysis and Flow Cytometry for Characterization of Endothelial Extracellular Vesicle Release. Int. J. Mol. Sci..

[57] Diehl P., Aleker M., Helbing T., Sossong V., Germann M., Sorichter S., Bode C., Mozer M. (2011). Incerased platelet, leukocyte and endothelial microparticles predict enhanced cpagulation and vascular inflammation in pulmonary hypertension. J. Thromb. Thrombolysis.

[58] Nanou A., Zeune L.L., Terstappen L.W.M.M. (2019). Leukocyte-Derived Extracellular Vesicles in Blood with and without EpCAM Enrichment. Cells.

[59] Italiano J.E., Mairuhu A.T.A., Flaumenhaft R. (2010). Clinical Relevance of Microparticles from Platelets and Megacaryocytes. Curr. Opin. Hematol..

[60] Yang K.S., Lin H.Y., Curley C., Welch M.W., Wolpin B.M., Lee H., Weissleder R., Im H., Castro C.M. (2020). Bead-Based Extracellular Vesicle Analysis Using Flow Cytometry. Adv. Biosyst..

[61] Wiklander O.P.B., Bostancioglu R.B., Welsh J.A., Zickler A.M., Murke F., Corso G., Felldin U., Hagey D.W., Evertsson B., Liang X.-M. (2018). Systematic methodological evaluation of a multiplex bead-based flow cytometry assay for detection of extracellular vesicle surface signatures. Front. Immunol..

[62] Stoner S.A., Duggan E., Condello D., Guerrero A., Turk J.R., Narayanan P.K., Nolan J.P. (2016). High sensitivity flow cytometry of membrane vesicles. Cytom. A.

[63] Lannigan J., Erdbruegger U. (2017). Imaging flow cytometry for the characterization of extracellular vesicles. Methods.

[64] Johnson S.M., Banyard A., Smith C., Mironov A., McCabe M.G. (2020). Large Extracellular Vesicles Can be Characterised by Multiplex Labelling Using Imaging Flow Cytometry. Int. J. Mol. Sci..

[65] Mykhailova O., Seghatchian J., Acker J.P. (2020). Assessment of extracellular vesicles using IFC for application in transfusion medicine. Transfus. Apher. Sci..

[66] Erdbrügger U., Rudy C.K.E., Etter M., Dryden K.A., Yeager M., Klibanov A.L., Lanningan L. (2014). Imaging flow cytometry elucidates limitations of microparticle analysis by conventional flow cytometry. Cytom. Part A.

